# Flipping the paradigm: a qualitative exploration of research translation centres in the United Kingdom and Australia

**DOI:** 10.1186/s12961-020-00622-9

**Published:** 2020-09-29

**Authors:** Tracy Robinson, Helen Skouteris, Prue Burns, Angela Melder, Cate Bailey, Charlotte Croft, Dmitrios Spyridonidis, Helena Teede

**Affiliations:** 1 Monash Centre for Health Research and Implementation, School of Public Health and Preventive Medicine, Monash University, Level 1, 43-51 Kanooka Grove, Clayton, Victoria, 3168 Australia; 2 School of Nursing, Midwifery and Indigenous Health, Charles Sturt University, Bathurst, NSW 2795 Australia; 3 Monash Partners Academic Health Science Centre, Clayton, Victoria Australia; 4 School of Management, College of Business, RMIT University, Melbourne, Australia; 5 Monash Health, Clayton, Victoria, Australia; 6 Warwick Business School, Gibbet Hill Road, Coventry, CV4 7AL United Kingdom

**Keywords:** Research Translation Centres, collaborations, metrics, workforce development

## Abstract

**Background:**

Over the past decade, Research Translation Centres (RTCs) have been established in many countries. These centres (sometimes referred to as Academic Health Science Centres) are designed to bring universities and healthcare providers together in order to accelerate the generation and translation of new evidence that is responsive to health service and community priorities. This has the potential to effectively ‘flip’ the traditional research and education paradigms because it requires active participation and continuous engagement with stakeholders (especially service users, the community and frontline clinicians). Although investment and expectations of RTCs are high, the literature confirms a need to better understand the processes that RTCs use to mobilise knowledge, build workforce capacity, and co-produce research with patients and the public to ensure population impact and drive healthcare improvement.

**Methods:**

Semi-structured interviews were conducted with selected leaders and members from select RTCs in England and Australia. Convenience sampling was utilised to identify RTCs, based on their geography, accessibility and availability. Purposive sampling and a snowballing approach were employed to recruit individual participants for interviews, which were conducted face to face or via videoconferencing. Interviews were recorded, transcribed verbatim and analysed using a reflexive and inductive approach. This involved two researchers comparing codes and interrogating themes that were analysed inductively against the study aims and through meetings with the research team.

**Results:**

A total of 41 participants, 22 from England and 19 from Australia were interviewed. Five major themes emerged, including (1) dissonant metrics, (2) different models of leadership, (3) public and patient involvement and research co-production, (4) workforce development and (5) barriers to collaboration.

**Conclusions:**

Participants identified the need for performance measures that capture community impact. Better aligned success metrics, enhanced leadership, strategies to partner with patients and the public, enhanced workforce development and strategies to enhance collaboration were all identified as crucial for RTCs to succeed.

## Background

The gap and time delays between evidence generation from research and translation into healthcare practice has been widely acknowledged [1, 2]. Despite considerable research investment, opportunities to translate new evidence across health systems are stymied when research is not embedded in clinical practice [3]. To date, a failure to engage relevant stakeholders as well as research and discipline ‘silos’ have contributed to barriers for knowledge translation and for the integration of research and education within healthcare [4, 5]. This fragmentation is highly evident in the divergent drivers and metrics employed across academic and clinical sectors to measure success. These and other factors hinder the achievement of a high quality, research-informed, sustainable and community-centred health system.

Over the past decade or more, Research Translation Centres (RTCs) have been established internationally in the United States, Canada, the United Kingdom and Australia. These centres aim to improve the integration of research, education and healthcare [5] and to overcome fragmentation. RTCs share a common purpose to accelerate the generation and translation of new evidence by fostering meaningful collaboration and integration between universities, health services and education providers [5] and to generate research and education that is responsive to health service and community priorities [3]. This approach extends the concept of ‘knowledge’ to include the experience of frontline clinicians and community members and has the potential to ‘flip’ traditional paradigms of education and research as the primary domain of ‘experts’, who, in the past, were seen as ‘sages on the stage’ [6, 7]. In contrast, RTCs aim to develop a paradigm of active participation and ‘community centric’ integrated healthcare, education and research. This is characterised by continuous engagement with stakeholders (especially service users, the community and frontline clinicians) to better understand priorities and health risks and to collectively generate priority knowledge to improve health outcomes [8, 9]. While RTCs offer a unique opportunity to drive an integrated, evidence-informed healthcare system, this endeavour requires a shift from traditional siloed research and poor translation, to a system where continuous collaborative learning and implementation become the focus.

Australia and the United Kingdom have world leading, universally accessible public healthcare systems. Both countries have actively invested in RTCs. In England, Collaborations for Leadership in Applied Health Research Centres (CLAHRCs) and Academic Health Science Centres and Academic Health Science Networks (AHSNs) have emerged. In Australia, similar entities have been established, with the National Health and Medical Research Council (NHMRC) accrediting seven Advanced Health Research Translation Centres (AHRTCs) (2015–2017) and three Centres for Innovation in Rural Health (CIRHs) [10]. RTCs in England have variable funding sources but, in Australia, they now receive substantive Federal Government funding via the Medical Research Future Fund (MRFF) (2018–2028) [11].

Although investment and expectations for RTCs are high, important evidence on how best to meet the vision and purpose of the RTCs and to translate and apply high quality research and evidence into practice is still needed [12]. The United Kingdom has a decade of experience with this new type of partnership. The Australian centres closely resemble the collaborative model adopted by the English CLAHRCs, with the third iteration of CLAHRCs funded in 2019 and re-branded as Applied Research Collaborations (ARCs). The ARCs have a focus on improving patient and public outcomes and undertaking clinical and applied research [9]. In Australia, RTCs are unique in that they are led by health services rather than by academic institutions. They also have a second starter advantage to learn from RTCs in England and internationally. Importantly, with unprecedented national collaboration through the Australian Health Research Alliance (AHRA) across all NHMRC-accredited centres, these RTCs also have the opportunity to learn from each other and to collaborate for system level changes. This is consistent with the literature that confirms a need to better understand the processes that RTCs use to mobilise knowledge, build workforce capacity and co-produce research with patients and the public to ensure population impact and to drive change and healthcare improvement [13, 14]. The primary aim of the current study was to explore particular dimensions of select CLAHRCs in England and RTCs in Australia in order to inform their ongoing progress and evolution in Australia. The questions that this research addressed included how ARCs and RTCs foster collaboration and how they address healthcare and community priorities. In addition, the research sought to understand the dimensions of RTCs, their leadership, strategies for collaborating with stakeholders (particularly communities and service users) and how they develop workforce capacity. These operational dimensions have been prioritised by the AHRA [15] and are explored here to inform the ongoing development of these promising new entities in Australia. Given the similarities between the ARCs in England and the AHRTCs and CIRHs in Australia, Academic Health Science Centres and AHSNs in the United Kingdom were not included.

## Methods

Qualitative, single semi-structured interviews (mean time of 1 hour) occurred with selected leaders and members from RTCs in England and Australia between April and August 2018. Convenience sampling was utilised to identify and recruit RTCs in England based on their geographic accessibility and availability at the time the interviews were conducted. Although not representative of all RTCs in England, the sample included one metropolitan and two regional centres in an effort to capture their diversity. In Australia, all RTCs that have been accredited by the NHMRC were available and recruited. More detail about participating RTCs appears in Table 1. In terms of individual participants, a purposive and snowballing approach was employed, whereby potential participants were identified by RTC leaders, early and mid-career researchers and, in the case of England, advisors from public and patient involvement (PPI) initiatives. Sampling was also influenced by thematic saturation. The rationale for this approach was based on identifying diverse participants who had both the knowledge and experience to describe the dimensions of interest explored in the interviews. At least two experienced qualitative researchers were present at each interview and interviewees were sent questions prior to the interviews. Researchers from the Monash Centre for Health Research and Implementation (Monash University) and the School of Business and Management (Warwick University) conducted the interviews. Collectively, they included PhD academics from diverse disciplines, including medicine, nursing, psychology and business. Interviewers had no prior relationships with participants but all were engaged in a wider programme of research designed to support the development of policy and practice to support the ongoing evolution of RTCs.

**Table 1:** Characteristics and foci of participating research translation centres

Research Translation Centre	Catchment characteristics and focus of Research Translation Centre
ARC 1	UK ARC serving a socio-economically and inter-generationally diverse region that includes a mix of urban and rural regions with substantial health inequalities.
ARC 2	UK ARC advantaged by a local agglomeration of biotech and health science organisations. Focal ambitions include clinical innovation and new technologies and therapeutics. Partners with local AHSN to facilitate spread of innovation and has interest in national as well as regional links and impact.
ARC 3	UK, ARC with focus on specific diseases and clinical conditions, together with cross-cutting enabling themes such as strengthening patient and service user involvement.
CIRH 1	Australian CIRH striving to build locally led research capacity to address entrenched health inequalities in local populations. Corresponding focus on social determinants of health. Strong community presence and influence over governance of the CIRH.
CIRH 2	Australian CIRH servicing regional and remote communities in an area roughly the size of England. Strong focus on community and patient voice, evident in co-design emphasis and education of researchers and clinicians in the process of implementation.
AHRTC 1	Urban-based, Australian AHRTC whose goals are less shaped by the specific needs of the local populace or by particular diseases, and more by research and capacity building for the future of medicine and care.
AHRTC 2	Urban-based, Australian AHRTC also without specific focus on the needs of the local catchment area. Core research streams are focused on specific diseases and health conditions, with effort also invested in mobilising expertise across organisational boundaries to advance research in these streams.
AHRTC 3	Australian AHRTC that is the single AHRTC in its region and therefore covers urban, regional and remote catchment areas. Its foci are informed by the health needs of its populaces and are on specific diseases and conditions.
AHRTC 4	Australian, urban-based AHRTC covering socio-economically diverse populations with strong focus on understanding and improving implementation, and developing enabling sciences (e.g. informatics) that cut across diseases and clinical conditions.
AHRTC 5	Australian, urban based AHRTC with significant reach in the greater region, and diverse communities. Strong focus on cross-cutting issues, rather than specific diseases, such as addressing health inequality and systems improvement.
AHRTC 6	Australian, urban based AHRTC with socio economically and culturally diverse populations in catchment area. Focus on dissolving boundaries to facilitate creativity and innovation; local impact is first priority, with clinical priorities determined by local need.
AHRTC 7	Australian, urban based AHRTC with strong heritage in precision health and data linkage, leading to global outlook and formation of global partnerships.
**Total:**	12

Key:

*ARC* Applied Research Collaboration

*CIRH* Centre for Innovation in Rural Health

*AHRTC* Advanced Health and Research Translation Centre

Potential participants were contacted via email or phone by members of the research team who were at ‘arms-length’ from participants. After receiving written consent from participants, interviews were conducted face to face or via videoconferencing between May and September 2018. All interviews were de-identified prior to broad thematic analysis. An interview guide was developed and included questions about how RTCs foster collaboration, address healthcare and community priorities, and seek to embed research and translation into healthcare at scale. In addition, participants were asked to identify the metrics they use to assess impact, their leadership, strategies for collaborating with stakeholders (consumer and community involvement), workforce development, and barriers to collaboration, as these areas have been prioritised in the literature [13, 16] and by the AHRA [17]. These questions extend the knowledge gleaned from our systematic and grey literature reviews on United Kingdom and Australian RTCs (Robinson T, Bailey C, Morris H, Burns P, Melder A, Croft C, et al. Bridging the research – practice gap in healthcare: a rapid review of research translation centres in the UK and Australia. Under Rev. 2020) and seek to address key knowledge gaps. Qualitative interviews were deemed the most suitable method for capturing and providing detailed insights into the complex operations of the RTCs. All interviews were audio-recorded, transcribed verbatim and analysed using a reflexive and inductive approach, described by Braun and Clarke [18] as the most appropriate for exploring a diversity of experiences.

Transcripts were read, re-read and coded. The coded data was reviewed to identify similarities and overlaps and the codes subsequently informed the generation of initial themes. A reflexive approach was adopted that involved two researchers comparing codes and interrogating themes that were reviewed and analysed inductively against the specific study aims and through meetings with the research team. In addition, notes taken during interviews were reviewed, which enabled further reflection on codes and themes. This study met the NHMRC priority for a healthcare improvement initiative and was therefore registered with the ethics committee but did not require ethics approval (H - #12480).

## Results

A total of 41 participants, 22 in England and 19 in Australia from a total of 12 RTCs (3 ARCs in England, 7 AHRTCs and 2 CIRHs in Australia) were interviewed. Given that the primary aim of the study was to inform the progress and ongoing evolution of RTCs in Australia, all accredited RTCs in this country were included.

Five major themes emerged from the interview thematic analysis, namely (1) dissonant metrics and drivers for healthcare improvement and research; (2) different models of leadership; (3) PPI and research co-production; (4) workforce development; and (5) barriers to collaboration. Quotes are presented as verbatim comments from participants and, where possible in terms of confidentiality, broad reference is made to their roles.

### Theme 1: dissonant metrics and drivers for healthcare improvement and research

Participants from both countries identified significant challenges in integrating applied healthcare and improvement approaches with more rigorous discovery and implementation research. Dissonant metrics between academic and healthcare sectors underpin this tension. Academic organisations largely rely on the traditional research metrics of grants, publications and citations, while healthcare measures focus more on service outcomes (length of stay, occasions of service and patient outcomes):

*“Yes we need to publish papers, but the* [health] *trusts are much more interested in whether we are actually improving service delivery, contributing to training, or whether we have actually implemented a quality improvement program that is of local relevance”* (Participant 19, Executive, ARC, England)

This dissonance was echoed by Australian participants:

“…*I think historically …we do basic science and then you look for a use for it and really it should be the other way around – we have got a clinical problem and how do we address that…*” (Participant 27, Executive, Australia)

This observation highlights how RTCs effectively need to have one foot in healthcare improvement and one in research, investing in healthcare improvement initiatives as well as traditional randomised studies. In the United Kingdom, local healthcare providers need service evaluations and rapid cycle improvement studies in their collaborations with the ARCs, but these research approaches are not always privileged by funding bodies or the academic sector, which often require probability-based research:

“…*Innovation does not have an evidence base to support it. It is about trialling and taking risks with new things. The academics need to have a whole rationale for why something would work.”* (Participant 13, Business academic, England)

Participants from Australia also identified challenges in ensuring health research is relevant to the needs of front-line staff:

“…*we weren’t interested in somebody developing an App or improving the specificity of a test. We wanted some change to a model of care that could be implemented straight away if it was shown to be useful.*” (Participant 37, Medical academic, Australia)

Being subject to traditional research metrics means that academics continue to undertake discovery research and clinical trials, which often comes at the expense of implementation and healthcare improvement studies that include processes for stakeholder engagement and co-production of research.

Although they were established to build multi stakeholder and co-produced research, in their first funding round, ARCs were primarily measured against traditional academic metrics. Funding criteria have since broadened but meant that ARCs that initially focused more on co-creation and collaborations faced particular challenges in demonstrating their impact with multiple stakeholders over the initial 5-year funding cycle:

“*They didn’t get the findings that were appropriate because it takes time … for that less mainstream model.*” (Participant 12, Business academic, England)

The use of traditional metrics was also a significant issue for the Australian RTCs:

“*The NHMRC may talk of translation* ...*translation isn’t really funded … there is still no incentive to do that.*” (Participant 32, Nurse academic, Australia)

However, participants did refer to the national AHRA alliance between RTCs and described how it was enabling “…*a clear framework that we can all be accepting nationally about knowledge translation and impact so we … don’t have a lot of duplicated effort or repetition when we are trying to report these things”* (Participant 29, Executive, Australia). The MRFF was identified as a potential enabler of applied research and translation, were “…*you could almost see the MRFF as an NIHR* [National Institute for Health Research, the largest funder of health research in the United Kingdom] *equivalent for Australia … with NHMRC at the discovery end and the MRFF in the middle, translation approach*” (Participant 29, Executive, Australia).

Participants from both countries identified difficulties in trying to demonstrate clinical and community impact when constrained and measured by traditional academic metrics. They also identified challenges demonstrating both national and local impacts:

“*For the national they need big studies, big papers, big impacts and yet their vision was that it* [the ARC] *would have local relevance.*” (Participant 21, Medical academic, England)

There is clearly a need to establish impact and success criteria that capture participation, collaboration and co-production, and are relevant to and prioritised by stakeholders and funders, but “…*how do we show government – like how do we collect the data that’s needed to show … we are being moved from the research front to the practice front?”* (Participant 20, Programme lead, United Kingdom). Ultimately, healthcare and academic metrics need to integrate and align to achieve a more holistic understanding and model of impact in both local healthcare improvement (a priority for health services) and larger transformational programmes and rigorous traditional evidence (favoured by researchers).

### Theme 2: different models of leadership

Participants from both countries concurred that the complex, cross-sector collaborations that RTCs seek to embed, and their mission to translate evidence into practice, deems leadership a crucial determinant of their success. In the English centres involved in this study, directors had academic backgrounds with demonstrated excellence in research that largely informed the centre’s strategic themes. Here, academic metrics may contribute to now largely discredited ‘top-down’ leadership approaches, where themes are developed and chosen according to a ‘push’ model, “…*not because they naturally hang together …but because the kind of CLAHRC period which started four years ago, wanted each theme led by an internationally recognised sort of research leader*” (Participant 17, Executive, England).

Participants affirmed a need to re-align this ‘top-down’ leadership approach:

“*It is very much top down and it’s quite interesting they actually got…leadership development programs to try and create distributed leadership. But then there still seems to be quite a lot of control from the top.*” (Participant 14, Mid-career researcher, England)

Distributive and collective models of leadership were identified as an important alternative, especially in Australia, where the RTCs are health service led:

“…*there’s no question in my mind that the most powerful leadership in these kinds of organisations are leaders who are able to work collaboratively with others – who’d distribute leadership.*” (Participant 29, Executive, Australia).

Some of the key personal qualities that support this leadership approach were identified:

“…*I don’t assume that I have the answers, so I’m there primarily as a facilitator and I respect the process and prioritised outcome and advocate for it, even if it’s not what I think is most important.*” (Participant 39, Executive, Australia)

Training needs to challenge the notion of leadership in hierarchies and instead focus on “…*handling institutional complexity … because you’re bringing together so many different parties and interests and their job is about being skilled as kind of like a conductor*” (Participant 29, Executive, Australia). The importance of leadership provided by middle managers was also emphasised:

“*Often the middle management are the people that set the strategy for the organisation and that actually facilitate a lot of the new collaborations….the Executives, are too busy … they’re away or they’re just engaged with other things – I think we could do with a middle* [layer of leaders] *… there isn’t really a next level*” (Participant 24, Executive, Australia).

This also applied to managers situated in health services:

“…*there hasn’t been enough focus on building the capacity of managers.*” (Participant 26, Executive, Australia)

On the basis of equity, the dominance of women in the healthcare workforce, and the preference of women for distributive leadership, participants identified an urgent need for “…*training in leadership and development of leadership in women*” (Participant 39, Executive, Australia), who are often operating in the middle level management of RTCs due to well-recognised barriers to career advancement and structural issues in medicine and healthcare that have yet to be overcome.

### Theme 3: PPI and research co-production

Participants were unanimous on the importance of stakeholder engagement, yet they identified that processes for genuine PPI (in United Kingdom terminology) or consumer and community involvement (CCI; in Australian terminology), were often lacking in research and healthcare improvement. There was consensus on the need to improve understanding of what PPI or CCI actually entail, what is most effective, and what processes are needed to guide meaningful and genuine co-production of research, translation and healthcare improvement with communities.

England has made significant advances at systems and grassroots levels, in progressing meaningful PPI, when compared with Australia, where system level changes are in their infancy and efforts remain fairly limited. In England, PPI advisors described wider health system level changes, including that PPI is mandatory for all research grant applications funded by the NIHR. Other English system level strategies that participants described for advancing PPI included frameworks and a collaboration with the NIHR to produce guides and conduct reviews that support PPI endeavours. The James Lind Alliance was cited as an example with a framework and toolkit for bringing together patients, carers and clinicians in priority-setting exercises to identify ‘treatment uncertainties’ and enable meaningful engagement. The James Lind Alliance is “…*a very specific method for making those joint priorities, but that’s only for a number of different areas* [treatment uncertainties]” (Participant 5, Programme lead, England). Another NIHR initiative described by participants was the Going the Extra Mile (Breaking Boundaries) Review [19], which identifies the characteristics of co-production and principles for PPI in healthcare research:

“…*we have very strong policy support at the highest levels and so when we did our Breaking Boundaries Review …. which was about reviewing the landscape nationally and internationally… we worked with the whole review panel to create recommendations which were very specific and are now being rolled out across the NIHR.*” (Participant 6, England)

All the ARC participants reported having PPI as separate or cross-cutting themes:

“…*the level of PPI within CLAHRCs is high …. We meet three times a year and we meet with the NIHR and we provide a report where we share what we’re doing.”* (Participant 6, England)

In England, PPI advisor positions are publicly advertised with clear role descriptions [19] and come with financial support, “…*which is 20 pounds per hour, 75 per half day, 150 pounds for a day and we are very transparent about what’s involved*” (Participant 6, England).

CCI was identified as crucial in Australia for the ongoing evolution of the RTCs as “…*consumers should be involved in every formal part of the structure*” (Participant 35, Executive, Australia). However, there were diverse views about what PPI in research and healthcare might actually look like or how consumers might have a role in the national research agenda:

“*The consumers are very focused on the local experience …our projects are often migrating into different contexts.*” (Participant 25, Executive, Australia)

While consumers have had limited involvement in healthcare planning and advocacy in Australia for the past decade, participants expressed a low awareness of how to co-produce research and no formal processes existed for recruiting or training CCI members. The question of how to ensure representative consumer voices was also raised:

“…*The challenge, of course, with community engagement is who are the right representatives.*” (Participant 41, Medical academic and clinician, Australia)

Australia has had very limited policy or funding incentives for CCI as well as limited training and experience, but system-, organisational- and individual-level strategies are now being prioritised nationally, with the RTCs designated a significant role. Indeed, the RTCs have come together nationally to create a CCI framework, establish priorities, undertake a national scoping exercise and co-design strategies, including training and a knowledge hub that will accelerate and deliver culture change in this area. In this activity, the Australian RTCs have drawn and learnt significantly from the experiences of ARCs in England.

In England, participants reported progress in measuring the impact of PPI. For example, the Guidelines for Reporting the Impact of Patient and Public Involvement in Research (GRIPP2) have been developed in England to improve the transparency and reporting of PPI impacts:

“…*they’re* [the NIHR] *keen to create a consistency of reporting.”* (Participant 6, England)

Narratives and case studies were also utilised in the United Kingdom to improve understanding of how PPI affects the experiences of patients, researchers and the public. The question of how to capture less formal impacts of PPI remains elusive:

“So*metimes someone will say to me that ‘I didn’t say anything in that meeting’ and I said … but that’s fine because the fact that you were there changed everybody’s way of thinking and so how you measure that …I don’t have the answers.*” (Participant 6, England)

Another challenge for PPI advisors related to the time-frames needed to develop research proposals and protocols:

“…*it takes the academics two months to get their heads around what they can do, what’s feasible”* (Participant 6, England)

Feedback to PPI leads from ARC members was also reported to be inconsistent and potentially tokenistic, raising questions about the authenticity of PPI in some instances:

“…*I was really shocked at the statistic of 50% of them* [PPI advisors] *being asked to be involved and never hearing anything again*…” (Participant 7, England)

Of more concern is the fact that *“…a lot of them had not even been told that the thing* [proposal] *they helped with had been funded”* (Participant 7, England). More work is needed to close the loop and establish true partnerships with patients, public, consumers and communities.

### Theme 4: workforce development

There was wide agreement among participants that the ability to implement and translate research evidence into practice and to strengthen collaborations between research, policy and practice requires specific skills and capabilities. Key themes for workforce development included a range of ‘global skills’ and the potential importance of dedicated roles – knowledge brokers (or dedicated translation roles).

In England, knowledge brokers were deployed in several ARCs or in partner organisations as intermediaries between academics and healthcare providers. Their roles and titles varied across ‘diffusion fellows’, ‘improvement fellows’ and ‘boundary spanners’. One participant explained that “*improvement science fellows are the navigators, while knowledge brokers facilitate diffusion*” (Participant 11, Early career researcher, England). In this context, knowledge brokers were seen as having a broader remit than improvement fellows, but their exact roles were somewhat blurred. Despite variation, knowledge brokers largely operated outside the organisational hierarchy and were seen as making a valuable contribution – especially in working across professional boundaries:

“*The people that emerged as being key in terms of having a brokerage role were the ones that had a hybrid background themselves.*” (Participant 22, Executive, England)

However, there was variation across ARCs in how these roles operated:

*“We had people from a range of quantitative and qualitative backgrounds, and a mix of clinicians and academic backgrounds, so they could mix their skills and projects and share their skills. These people are in demand.”* (Participant 19, Executive, England)

However, diffusion fellows and knowledge brokers did not always have clinical ‘credibility’:

“…*they are relatively junior coming into the world and a setting that is highly professional.*” (Participant 21, Executive, England)

The Fellows themselves acknowledged challenges with their bridging roles, especially the tension between the independence and isolation of the research-related dimensions of their role, and the inherently interdependent nature of knowledge brokering:

“…*I deal with the clinicians and they ask me about the research … but it’s my research not our research …*” (Participant 14, Mid-career researcher, England)

They also identified tension between the independence and isolation that their role entails:

“*You do have the independence of your time* [but] *you are quite isolated and it’s like doing the PhD which is very, you know, your project.*” (Participant 14, Mid-career researcher, England)

Another concern was a lack of clear career progression/paths for knowledge brokers:

“…*we need to be moving upwards and developing sort of middle grade faculty appointments because obviously we now have quite significant numbers of these people who have come through our CLAHRC.*” (Participant 21, Executive, England)

Career progression was a particular issue for knowledge brokers situated in clinical settings. The need for a critical mass or ‘army’ of knowledge brokers to ensure their work is sustained was identified, as was their role as change agents:

*“They need to want to change things. They need to be in the boat and rock the boat, but they can’t fall out of the boat. They have got to do it from within, embedded in the community but disposed towards change.”* (Participant 14, Mid-career researcher, England)

In Australia, universities have traditionally developed academic chair roles – positions funded by both academia and health services to provide leadership in research and clinical care and to drive integration. Participants identified a greater need for a “…*hybrid clinician that leads and owns the research, but has got a strong set of academic credentials that we’ve* [the AHRTC] *helped develop*” (Participant 28, Executive, Australia). In Australia, there has been little to no funding for health services, implementation or applied researchers (<5% of NHMRC funding), with a major gap in the workforce and skills. The funding models have also focused in the past on traditional research metrics, which make it difficult for active clinicians to compete with full time academics. Whilst this is changing, participants expressed concerns about the challenges experienced by clinical academics in this context:

“…*I think we’ve lost a bit of ground…they are doing two impossible jobs, they are trying to be a good clinician and trying to be a good change agent and researcher … we are losing clinical academics.*” (Participant 32, Nurse academic, Australia)

In terms of skills, English participants identified programme and service evaluation as crucial workforce capabilities for RTCs. This includes more than traditional programme logic approaches or the efficacy and effectiveness paradigms of implementation research. Participants identified implementation frameworks such as RE-AIM and the Consolidated Framework for Implementation Research (CFIR) but also reported a low awareness and skills in these methods among both clinical and academic staff.

Global skills such as digital health, communication, team-work, priority-setting and leadership were also deemed important:

“*You need the communication skills, you need the leadership skills to influence people, you need to listen to the local authorities, and you need the technical skills to get the evidence.”* (Participant 15, Business academic, England)

Diplomacy skills, such as the ability to build and maintain positive relationships, link and network with others, and deal with conflict effectively, were important for maintaining partnerships with local authorities, governments, stakeholders and end-users. Additionally, qualities such as “…*emotional intelligence, the empathy, the interest in those around them”* (Participant 11, Early career researcher, England) were identified as important, as were team based models of care and project management.

Participants from one English ARC described their programme of education and workforce development on implementation, which includes both accredited academic courses and more tailored workforce programmes for clinicians. Implementation and improvement knowledge were embedded in all their themes, with a discrete cross-cutting theme in implementation science that enabled them to develop a suite of tailored education programmes. These programmes included a Master in Implementation and Improvement Science, a 3-day Masterclass and whole day meetings with PPI representatives to clarify processes for identifying gaps and research priorities. Flagship programmes, including mental health, were identified and initiated:

“…*we realised we needed to be offering a number of things.*” (Participant 21, Executive, England)

In Australia, with a more limited workforce in applied research, participants tended to cite traditional research skills as important:

“…*we run a whole program of courses on cost-effectiveness, on implementation, on study design, on statistics, meta-analysis*…” (Participant 25, Executive, Australia)

However, the need for workforce development programmes to also address informal or global skills, such as negotiating and communication, was recognised:

“…*the intangibles, professionalism, patient safety, patient experience – professionalism is a big topic …how doctors interact with themselves and their patients.*” (Participant 41, Medical academic and clinician, Australia)

Overall, there was consensus that workforce development programmes need to be tailored and that traditional unidirectional education alone is insufficient in the absence of opportunities and motivation to apply new skills in clinical settings:

*“What is the right approach to upskill people? It is not just sitting in a lecture theatre and giving them knowledge. You need to help them absorb this knowledge by taking it into their environment.”* (Participant 15, Business academic, CLAHRC, England)

This comment highlights the importance of integrating education and learning into healthcare:

*“It is more than workshops. It is situated learning. It is about them doing real life projects, leading them, and learning as they go along.”* (Participant 14, Mid-career researcher, England)

The United Kingdom experience is instructive in terms of recognising how partnerships between the ARCs and health services have facilitated opportunities for applied learning and the testing of new interventions:

“…*most of them work as incubators, so early incubators or later stage incubators.*” (Participant 22, Executive, England)

There is significant potential, therefore, for RTCs to operate as ‘test beds’ for implementation and healthcare improvement:

*“It is about the ability to experiment, learn from mistakes, reflect, lead, and share collective responsibility.”* (Participant 21, Executive, England)

### Theme 5: barriers to collaboration

All participants identified that collaboration was fundamental for RTCs in both England and Australia. However, in England, participants were more likely to identify collaboration as a key endeavour. In Australia, where RTCs are health service led and governance includes both health services and academic institutions, participants saw collaboration as integral to their structure and had a stronger focus on a ‘translation’ mission, consistent with policy and funders’ positioning of these entities. In England, “…*it’s not about translation of existing research but about development of partnerships between a variety of stakeholders in order to find ways to improve health…*” (Participant 20, Programme lead, England).

However, a number of barriers to collaboration were identified. In England, ARCs have a 5-year cycle of funding that presented some concerns as to whether collaborations could be sustained:

“…*the funding cycle can disrupt relationships as they start to get established.*” (Participant 8, Executive, England)

Participants also described how ARCs are annually ranked against each other often on traditional metrics:

*“We are not supposed to be competing against each other, but then when we submit the annual report … they rank us all, and everyone knows what their number is.”* (Participant 4, Medical academic, England)

The focus on traditional academic metrics and previous funding models meant that, initially, genuine collaboration was not always rewarded:

*“Collaborating across CLAHRCs is not a priority because you are all competing for the same pot of funding.”* (Participant 8, Executive, England)

Even collaboration within ARCs is challenging:

“…*the sheer practicalities of it. Someone’s got to get from A to B.*” (Participant 9, Medical academic, England)

The need for more national collaboration between ARCs was identified as crucial in any future iterations:

“…*you could have your individual CLAHRCs but you could have certain national themes like workforce … as priorities. And each CLAHRC would have to commit a certain amount of funding to those national priorities.*” (Participant 10, Medical academic, CLAHRC, England)

One participant commented on the irony that, “…*in some ways it might be easier to have an international network rather than UK based one, because one of the challenges in the UK is they compete for the same funding*” (Participant 12, Business academic, England).

In Australia, there was evidence of direct learnings from England and attempts to avoid direct competition. Here, the accreditation by NHMRC (based on meeting criteria and not on competition across RTCs), the strong national collaboration afforded by the AHRA and the agreement to award equal funding by the MRFF to all NHMRC-accredited RTCs, has created a system whereby RTCs can genuinely collaborate and avoid competition. This has also allowed the centres to jointly establish a range of national priorities, has facilitated collaboration across centres, reduced competition and duplication, and accelerated sharing and the impact of the RTC work:

“…*collaboration from the national alliance of the nine centres has been remarkable…when we got our funding this year every centre director just said we will commit to continuing to implement these national frameworks*…” (Participant 28, Executive, Australia)

However, there was acknowledgement from some participants that a certain level of competition might continue and that centres would need to identify where there was most value in collaboration. National system-level initiatives include “… *setting up a national data record and sharing – agreeing on a set of privacy principles … but at a state level there are things …where we’ll compete*” (Participant 26, Executive, Australia).

Participants in Australia expressed a strong commitment to the AHRA and willingness to strengthen their national collaboration:

“…*there is a mechanism to firstly find out what’s going on across the other sites … we can be strategic.*” (Participant 28, Executive, Australia)

In addition, the alliance was identified as a key factor in improving engagement with government:

“…*when we are having those individual centre discussions with governments and our own state governments we actually have come together and shared some of the principles, difficulties and communications so we can actually then go back with a shared voice.*” (Participant 37, Executive, Australia)

Participants reported that the AHRA has convened national steering committees (to address system-wide priorities) and different RTCs are working together to advance shared knowledge around these priorities. These include (1) data-driven healthcare improvement, (2) CCI, (3) clinical research facilitation, (4) Indigenous capacity-building, and (5) health services research and workforce development [17]. Indeed, multiple participants maintained that AHRA has been the most significant enabling factor for collaboration and for the acceleration and sharing of learning across centres in Australia:

“*It’s open discussion, decision-making and the delivery of the national frameworks …when we got our funding this year every Centre Director just said we will commit to continuing to implement these national frameworks…*” (Participant 42, Executive, Australia)

In England, high staff turnover was a factor identified as a barrier to collaborative relationships. The National Health Service (NHS) appears subject to political changes; attempts to significantly reform the NHS are common and frequent. Even at a regional or organisation level, this was identified as a significant challenge. Participants described how the NHS has experienced a number of leadership changes in recent years and this creates significant disruption, as researchers need to continually re-establish and re-strengthen their relationships:

*“The NHS is constantly changing, and people move on so quickly there. The four main NHS trusts with whom we work now have different chief executives and different people on their boards.”* (Participant 22, Executive, England)

Staff attrition also impacts on priority-setting processes:

*“Although we have annual stakeholder meetings, we hardly ever get the same audience. It’s always a different group of people.”* (Participant 20, Programme lead, England)

The United Kingdom experience also highlighted structural barriers to collaboration, whereby ARC themes can reinforce internal silos:

*“We are always trying to cross-refer and think about how all of the themes are working and whether aspects of what one theme does can have an impact on another. But often it doesn’t happen because they are quite specialised.”* (Participant 4, Medical academic, England)

Relationships between leaders and their partners were crucial factors that enabled collaboration. In England, “*what makes the difference is that the leaders are willing to put in the time and agree to communicate with our partners, spending many hours at coffees, dinners, lunch, and breakfast meetings to slowly build those relationships so you can get to the stage of trusting each other and understanding our visions”* (Participant 8, Executive, England). The emphasis on the importance of relationships highlights the challenge but also the importance of capturing more informal types of communication as key enabling factors for collaboration:

*“We talk to our AHSN lead on a weekly basis, it is the pick-up-the-phone-and-ask-a-question type of relationship that makes a huge difference.”* (Participant 10, Medical academic, England)

There is a need to ‘unpack’ what these collaborations actually entail and how their impact and shape may be captured:

“...*what will these collaborative practices look like, how do we actually even, where do we begin …you know to unfold that.*” (Participant 13, Business academic, England)

## Discussion

In Australia, the complex system of government and healthcare funding both impedes change and protects the health system from political interference. Because State Governments fund and administer hospitals while the Federal Government is responsible for primary and ambulatory care, political interference in healthcare can be more difficult. A core aim of RTCs in both Australia and the United Kingdom, as leading universal public health systems, is to drive better health outcomes for the population by embedding research and education in healthcare and accelerating the translation of new evidence into practice. This study sought to gain an in depth understanding of strategies to deliver on these aims by examining metrics and drivers, leadership, PPI, research co-production, workforce development, and collaboration. Key stakeholders, including RTC leaders, community and consumer representatives, and early- and mid-career researchers, participated in semi-structured interviews, revealing five broad themes for addressing the evidence-practice gap and integrating research and education with healthcare – dissonant metrics, different models of leadership, stakeholder engagement, workforce development, and barriers to collaboration.

Participants from RTCs in both England and Australia identified dissonant metrics with a tension between traditional academic metrics of publications and citations while healthcare is measured by service outcomes such as occasions of service and length of stay. In addition, they reported the challenge of trying to balance local healthcare improvement studies with large-scale implementation research. Not all clinical problems or gaps in care are amenable to sophisticated implementation studies and healthcare providers may have a greater need for service and programme evaluations (often within short time frames). Given that health services often require pragmatic studies that produce rapid impacts on patient care, there is arguably a need to ‘move’ researchers from traditional discovery research towards more service evaluation and healthcare improvement that has clinical relevance.

However, there are also concerns that potentially less rigorous healthcare improvement methods need to be strengthened [20]. The scope of implementation research is broad and rigorous, with studies that seek to understand evidence-practice gaps, proof of concept studies, large scale implementation and effectiveness trials of complex interventions [21]. Participatory research processes are necessary to ensure that research aligns with the needs of services and communities and that processes for co-production are established. Such an approach relies more on ‘lived experience’ than science and raises questions about how to increase the rigour and trustworthiness of participatory evaluations. Traditional rigorous clinical investigation, such as randomised controlled trails, focus on efficacy in highly selected populations yet implementation research focuses on broad population cohorts and contexts that encompass implementation and effectiveness [21]. Both approaches are important to embed the translation of evidence into practice. Hence, both healthcare improvement and implementation science need to better align with community needs in order to have more legitimacy and it is crucial that healthcare improvement acquires a more robust evidence base and becomes more of a collective endeavour [20].

Leadership was a common theme reported by participants in RTCs. Two important aspects of translation identified by participants were how to apply and how to scale up new evidence – both of which were reported to be facilitated by distributed models of leadership. Despite evidence that effective leadership in healthcare correlates with quality care and improved patient outcomes [22], few participants in this study had received formal training in leadership. The concept of leadership as a collective and social process where employees are empowered to work collaboratively and take on leadership roles is likely to prove difficult in the healthcare sector, where hierarchical structures and political influence combine to make effective change hard to achieve [23]. Despite these difficulties, clinicians need to drive improvements and have greater accountability for quality improvement if we are to respond effectively to the growing complexity of healthcare [24]. The challenge of how to integrate research in healthcare is arguably not amenable to hierarchical leadership. Distributed leadership offers a means of harnessing insights from multiple stakeholders and improving the quality of decision-making [25]. Study findings accord with current literature on leadership and several participants identified that distributed and collective leadership need to be embedded and evaluated in RTCs. Interestingly, leadership training has often become the bastion of business and corporate endeavours, costs can be prohibitive, and co-design and evaluation limited [26]. In addition, a greater focus on leadership training for women in healthcare improvement has been highlighted in the literature [27].

PPI, or CCI, emerged as an important theme and was identified by participants from both the United Kingdom and Australia. There was wide agreement that CCI is crucial at all stages of translational research and healthcare improvement. Whilst the English RTCs appear more advanced in terms of national strategies, system level initiatives such as grant funding requirements and guides to processes, gaps were identified. Questions persist about the extent of PPI required for projects, the authenticity of PPI in some instances, and how to fund and embed it as core business. ARCs all identified PPI as an important strategy but were still grappling with whether and how to embed PPI in their structures and processes. In Australia, so called CCI is relatively under-developed, with some work emerging at a national level across the RTC Alliance (AHRA) and in funding body requirements. Nevertheless, genuine PPI partnership in the research agenda of RTCs is a priority and appears to be progressing in both countries, presenting an important opportunity to study and further understand optimal processes and impact.

In England, participants noted that the partnerships that ARCs have with healthcare (the NHS) have enabled clinical settings to operate as ‘test beds’ for new innovations and implementation studies. This finding highlights how stakeholder engagement needs to be multi-level with input from healthcare providers. When we think of stakeholder engagement, we often immediately think of consumers/patients and the community. Here, we show that, in healthcare improvement, front-line clinicians and health service managers are key stakeholders in building a culture of improvement. How services involve those who provide direct patient care in the planning, delivery, improvement and evaluation of health services was identified as a significant determinant of the uptake of innovations. Information from the interviews also highlighted that health practitioners require data that relates to the lived experience of patients as well as access to training on basic improvement skills (these are not widely taught in current undergraduate or workforce development programmes). Again, research and evidence translation on how to optimally engage with and build the capacity of clinicians remains a key gap.

Workforce development was another core theme for RTCs as an important enabling factor to support the integration of research and education in healthcare. In England, a suite of tailored workforce development programmes and accredited academic courses in implementation have been developed in one ARC, but participants stated that it is important to ‘marry’ these approaches with healthcare and quality improvement methods to ensure their clinical relevance. Global skills, including stakeholder engagement, influencing skills, systems thinking, negotiating and team work, have all been identified as significant workforce capabilities for enabling a culture of learning and improvement [28]. Participants in the current study confirmed the importance of ‘global skills’, such as communication and active listening, but also in relation to fostering collaborations between universities and healthcare providers. In this context, participants identified healthcare leaders (and middle managers) as a key target group for workforce development. In England, new hybrid roles have been deployed to bridge the divide between universities and health services; this approach has also been trialled in Canada. However, in both countries, these roles are in their genesis and questions remain about career progression, possible isolation and efficacy. The English participants also noted a need for a critical mass of ‘boundary spanners’ and research savvy practitioners to engage and support this. In Australia, a dearth of skills in applied research was noted, with limited funding and a past focus on traditional research skills. Development of this workforce is now a priority under the AHRA system-level initiatives and is a focus of national collaboration with shared and freely accessible online programmes and internal capacity-building activities now in place. RTCs in both countries have the potential to harness the experience and knowledge of frontline clinicians, the community and consumers, with researchers able to facilitate learning on how to implement new knowledge across different settings. Workforce development in applied research skills are a priority and extend beyond clinical or research skills to global skills.

Regarding collaboration, whilst RTCs have significant potential to integrate research, education, and healthcare and to overcome silos, barriers were identified. New approaches are needed to enable collaboration and align RTC processes and objectives. Participants noted system barriers to collaboration, including competition, dissonant metrics, and unclear measures of impact and success. These threaten to derail the collaboration and integration required for RTCs to succeed. In England, early funding schemes and traditional metrics exacerbated the tension between sectors and sustained competition, despite collaboration being central to RTC endeavours. Processes for demonstrating impact of the ARCs have evolved since their inception but, despite an aspiration to strengthen national collaborations, there is currently no national structure or funding for this endeavour in England. In Australia, system strengths include health service leadership of the centres and independent NHMRC accreditation of centres that is not linked to funding. While accreditation is competitive once this is achieved, funding is equally shared across Australian RTCs and facilitated through the national alliance. Whilst, in many areas, Australia is less advanced and mature than England, the need to build a national alliance and avoid competition was recognised after observing some competition across RTCs in England and is an example of a ‘second mover advantage’. As a result, AHRA has enabled collaboration and is able to focus on the goal of integration of research and healthcare, and translation, rather than the removal of barriers to collaboration. The national alliance of RTCs, for example, has facilitated the development of shared, high-level health and system priorities in five areas: healthcare service research, data-driven healthcare improvement, Indigenous health, clinical research and workforce development. AHRA has also facilitated processes for collaboratively identifying and defining problems or gaps in care through multiple stakeholder lenses. There may be potential learnings applicable to the English setting from the systems developed in Australia that facilitate shared learnings, avoid duplication and competition, and facilitate collaboration.

Since RTCs have been established and funded for research and translation, performance measures around impact are needed. Little evidence emerged during the interviews on optimal impact measurements and strategies. Narratives or case studies have been used [29], whilst the NHMRC in Australia is using reports based on evidence of moving research along the ‘translation impact pathway’ at the system, process and project level [29]. In England, the Research Excellence Framework has focused and linked university funding to impact. Australia is yet to implement system-level changes that generate success metrics that are aligned to funding. Optimal strategies to measure impact are needed internationally in the context of RTCs.

The limitations of this study must be acknowledged, including reliance on purposive and convenience sampling in England that was vulnerable to selection bias with only a subgroup of English RTCs participating in interviews. Future research should engage broader ARCs as they have continued to evolve. Nevertheless, this study allowed for in-depth exploration and understanding of RTCs and a comparison across a more mature English system and an emerging Australian system, both of which operate in the context of leading public universal health systems. RTCs have received significant investment. Accordingly, expectations are high that they will integrate research, education and healthcare, build collaboration, and deliver health impact into the future. This finding highlights that, although there is a mandate or focus on ‘education’ across RTCs, it remains difficult to elucidate clearly who the target is and to what end and how this is differentiated from other education bodies. An area for future focus for the RTCs is to identify the niche where gaps are identified, added value can be provided and duplication avoided.

Advances are occurring; however, further development is needed, including better aligned success metrics, enhanced leadership, more mature strategies to partner with patients and public, enhanced workforce development, and strategies to enhance collaboration such as the AHRA and removal of direct competition in Australia. Finally, there is a need to clarify the optimal approaches for measuring the impact of RTCs.

## Data Availability

The datasets used and/or analysed during the current study are available from the corresponding author on reasonable request.

## Abbreviations

AHSC: Academic Health Science Centre
AHRA: Australian Heath Research Alliance
AHRTCs: Advanced Health Research Translation Centres
AHSNs: Academic Health Science Networks
ARCs: Applied Research Collaborations
CLAHRCs: Collaborations for Leadership in Applied Health Research Centres
CCI: consumer and community involvement
CIRHs: Centres for Innovation in Rural Health
MRFF: Medical Research Future Fund
NHMRC: National Health and Medical Research Council
NHS: National Health Service
PPI: public and patient involvement
RTCs: Research Translation Centres
